# *CYP1A2* rs762551 polymorphism contributes to cancer susceptibility: a meta-analysis from 19 case-control studies

**DOI:** 10.1186/1471-2407-12-528

**Published:** 2012-11-19

**Authors:** Hongge Wang, Zhi Zhang, Sugui Han, Yujuan Lu, Fumin Feng, Juxiang Yuan

**Affiliations:** 1 Department of Epidemiology, College of Public Health, Hebei United University, Tangshan, 063000, China; 2 Department of Cancer, Chemotherapy and Radiology, Tangshan Gongren Hospital, Tangshan, China; 3 Department of Clinical laboratory, Tangshan Renmin Hospital, Tangshan, China

**Keywords:** *CYP1A2*, Cancer, Meta-analysis, Polymorphism

## Abstract

**Background:**

Genetic polymorphism (rs762551A>C) in gene encoding cytochrome P450 1A2 (*CYP1A2*) has been shown to influence the inducibility of *CYP1A2* expression and thus might be associated with risk of several types of human cancer. However, the results of previous studies on the associations of this polymorphism with risk of cancer are not all consistent. To clarify the potential contribution of *CYP1A2* rs762551 to cancer risk, we performed a meta-analysis of the published case–control studies.

**Methods:**

We used PubMed, Embase, OVID, ScienceDirect, and Chinese National Knowledge Infrastructure databases to identify the related publications for this meta-analysis. The pooled odds ratio (OR) and 95% confidence interval (CI) were calculated using random effect model to evaluate the association of rs762551 with cancer risk. A *χ*^2^-based Q-test was used to examine the heterogeneity assumption and the funnel plot and Egger’s test were used to examine the potential publication bias. The leave-one-out sensitivity analysis was conducted to determine whether our assumptions or decisions have a major effect on the results of the review.

**Results:**

Our analysis of 19 eligible case–control studies showed a significant association between rs762551C variant with risk of cancer in the genetic model of CC versus AA (OR = 1.30, 95% CI = 1.02-1.64) and the dominant model (OR = 1.19, 95% CI = 1.04-1.36). In subgroup analysis based on ethnicity, the rs762551CC genotype was associated with increased cancer risk (OR = 1.29, 95% CI = 1.27-1.63 in co-dominate model and OR = 1.17, 95% CI = 1.02-1.34 in dominant model in Caucasians, but not in Asians and the mixed population.

**Conclusion:**

These results suggested that *CYP1A2* rs762551 polymorphism is likely to be associated with susceptibility to cancer in Caucasians.

## Background

The cytochromes P450 (CYPs) played an important role in the development of various cancers, since they involved in the metabolic transformation of numerous endogenous and exogenous compounds including carcinogens and anti-cancer drugs [1]. Cytochrome P450 1A2 (CYP1A2), as one of important CYP enzymes, is responsible for the metabolic activation of pro-carcinogens such as heterocyclic aromatic amines (HAA), polycyclic aromatic hydrocarbons (PAHs) and 4-methylnitrosamino-1-(3-pyridyl)-1-butanone (NNK) and further contributes to the risk of cancer [2,3].

*CYP1A2* gene has been mapped on chromosome 15q24.1 and head to head shares a bidirectional promoter with *CYP1A1* gene [4]. It has been demonstrated that the expression of *CYP1A2* existed large inter-individual variability in the liver [5] and it is believed that the expression of *CYP1A2* is regulated by constitutive expression and inducible expression from environmental chemicals [3,6].

*CYP1A2* is highly polymorphic and there are more than 200 polymorphisms existed in *CYP1A2* gene region according to NCBI dbSNP database ( http://www.ncbi.nlm.nih.gov) and SNP500Cancer ( http://variatgps.nci.nih.gov). Previous studies have extensively focused on *CYP1A2* -164A>C polymorphism (CYP1A2*1F; rs762551) [7,8], which is located in the intron1 of *CYP1A2*. There also have several reported studied three common variations, that is -3860G>A polymorphism (CYP1A2*1C; rs2069514) [8,9], -739G>T polymorphism (rs2069526) [8,10] and 1545T>C polymorphism (rs2470890) [10,11]. These polymorphisms may be related to altered inducibility of CYP1A2 expression by environmental chemicals and consequently influence the individual susceptibility to certain cancer. For example, the *CYP1A2* rs2069514 A allele has been demonstrated to associated with decreased enzyme activity in smokers and *CYP1A2* rs762551 polymorphism is also associated with altered expression of *CYP1A2* by cigarette smoking [12-14].

Recently, many studies have investigated the association of *CYP1A2* polymorphisms and the risk of various cancers, including lung cancer, breast cancer, colorectal cancer, stomach cancer and others in various populations [15-18]. However, the results of these studies are not consistent and inconclusive. Considering the role of CYP1A2 in defending against environmental carcinogens and in the development of cancers, we performed a systematic meta-analysis from all eligible studies to address the overall risk of *CYP1A2* variants in the development of all cancers involved.

## Methods

### Identification and eligibility of relevant studies

Remote PubMed, Embase, OVID, ScienceDirect, and Chinese National Knowledge Infrastructure database (between January 2003 and December 2011) was searched using the search terms: CYP1A2/P4501A2/phase II enzymes, polymorphism/polymorphisms/genotype and cancer/carcinoma/adenomas to identify all publications, which investigated the association of the *CYP1A2* polymorphism with cancer risk in all ethnic populations. We evaluated the titles and abstracts of all relevant papers, but excluded case reports, editorials and reviews. All publications in English language with available full text matching the eligible criteria were retrieved. For inclusion in this meta-analysis, the identified articles had to provide information on the following: (1) using a case–control design, (2) sufficient data for examining an odds ratio (OR) with 95% confidence interval (CI) and (3) was a study of the *CYP1A2* rs762551 polymorphism and cancer risk, (4) genotype distributions of polymorphism are consistent with hardy-Weinberg Equilibrium (HWE). In addition, we checked the references of relevant reviews and eligible articles that our search retrieved by two investigators independently.

### Methods for quantitative analysis

We examined the association between *CYP1A2* rs762551 A>C polymorphism and the risk of cancer by calculating pooled odds ratio (ORs) and 95% confidence intervals (CI) in genetic model of CC versus AA, dominant model (CC+CA versus AA) and recessive model (CC versus CA+AA). The significance of pooled OR was tested by Z test. The *χ*2-based Q-test was also used to examine the heterogeneity assumption [19]. If studies’ findings only differ by the sampling (*P*≥0.05), a fixed-effects model could be used to calculate the combined OR. By contrast, if the *P* value of the Q tests is below 0.05, which showed that the study results statistically differ by heterogeneous case and sampling, a random-effects model could be more suitable. Since we used accumulating data from a series of studies, which had been conducted by researchers operating independently, the random model was more easily justified than the fixed model [20,21]. The summary OR and 95% CI were calculated under the random effect model.

The leave-one-out sensitivity analysis was conducted to determine whether our assumptions or decisions have a major effect on the results of the review by omitting each study (one at a time) [22]. Furthermore, subgroup analyses were performed to test whether the effect size varied by the ethnicity and the source of control population. To evaluate the published bias, we used funnel plot analysis, which is graphical display of sample size plotted against effect size for the studies included in a meta-analysis [23]. To test for funnel plot asymmetry, Egger’s test was performed [24]. All of calculations were performed using R program.

## Results

### Characteristics of meta-analyses database

**Figure 1:**
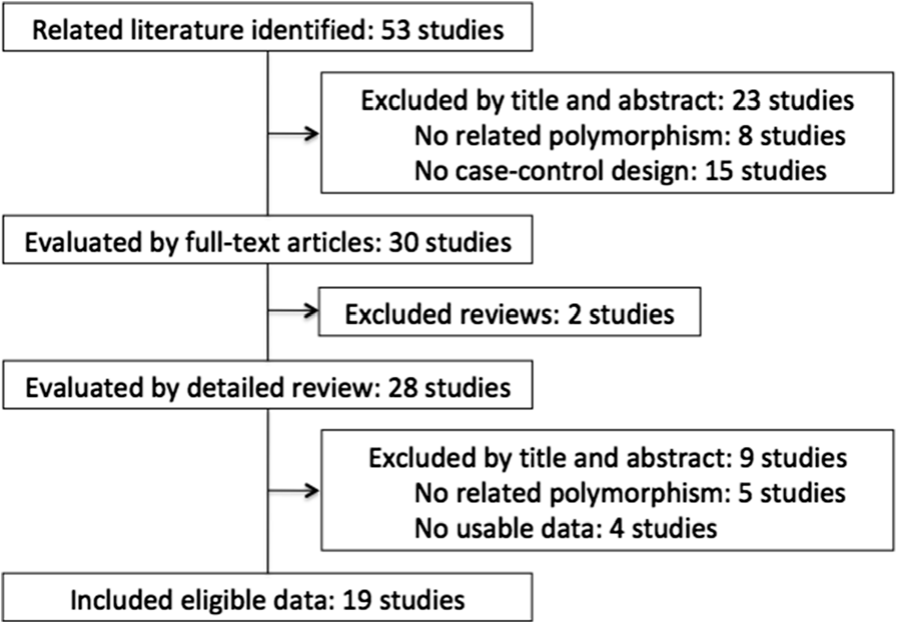
The flow chat for study identification.

After preliminary screening as of 15 December 2011, there were 53 relevant publications fitting the key terms. We excluded 34 studies by (1) no related *CYP1A2* polymorphism, (2) no cancer case–control design, (3) review articles [25,26], (4) no usable genotype data [27-30] and included 19 studies [7-11,16-18,31-41] in this meta-analysis (Figure 1 and Table 1). Overall, the studies involved in 8,218 cases and 11,165 controls. The genotype distributions for *CYP1A2* rs762551 polymorphism are shown in Table 2.

**Table 1:** Characteristics of the studies included in the meta-analysis

**Author**	**Year**	**Country**	**Ethnicity**	**Cancer type**	**Cases**	**Controls**	**Source of controls**	**Genotype method**
Khvostova [7]	2011	Russia	Caucasian	Breast	323	526	Hospital	PCR-RFLP
MARIE-GENICA [31]	2010	German	Caucasian	Breast	3147	5485	Population	PCR-RFLP
Singh [8]	2010	India	Asian	Lung	200	200	Population	PCR-RFLP
Sangrajrang [16]	2009	Thailand	Asian	Breast	552	483	Hospital	TaqMan
B′chir [9]	2009	Tunisia	Tunisian	Lung	101	98	Hospital	PCR-RFLP
Kobayashi [17]	2009	Japan	Asian	Stomach	141	286	Hospital	MassARRAY
Altayli [32]	2009	Turkey	Caucasian	Bladder	135	128	Hospital	PCR-RFLP
Aldrich [33]	2009	USA	Mixed	Lung	113	299	Mixed	Not defined
Saebo [35]	2008	Norway	Caucasian	Colorectal	198	222	Hospital	PCR-RFLP
Suziki [34]	2008	USA	Caucasian	Pancreatic	649	585	Population	PCR-RFLP
Yoshida [36]	2007	Japan	Asian	Colorectal	64	111	Hospital	PCR-RFLP
Osawa [18]	2007	Japan	Asian	Lung	103	111	Hospital	PCR-RFLP
Gemignani [10]	2007	Italy	Caucasian	Lung	297	310	Hospital	Microarray
Kotsopoulos [37]	2007	Canada	Caucasian	Breast	170	241	Hospital	PCR-RFLP
Bae [40]	2006	Korea	Asian	Colorectal	111	93	Hospital	PCR-RFLP
Long [38]	2006	China	Asian	Breast	1082	1139	Population	PCR-RFLP
Li [39]	2006	USA	Caucasian	Pancreatic	307	333	Population	PCR-RFLP
Landi [11]	2005	spain	Caucasian	Colorectal	361	321	Hospital	APEX
Goodman [41]	2003	USA	Caucasian	Ovarian	164	194	Population	PCR-RFLP

*PCR*, polymerase chain reaction; *RFLP*, restriction fragment length polymorphis; *APEX*, arrayed primer exension.

**Table 2:** CYP1A2 rs762551 polymorphism and cancer risk stratified by characteristics of studies

**Variables**	**Genotype of rs762551 (Cases/Controls)**				**CC vs AA**	**Dominant model (CC+CA vs AA)**	**Recessive model (CC vs CA+AA)**
	**Total**	**AA**	**AC**	**CC**	**OR (95% CI)**	**OR (95% CI)**	**OR (95% CI)**
All	8218/11165	3750/5374	3567/4720	901/1071	1.30(1.02-1.64)*	1.19(1.04-1.36)*	1.19(0.99-1.44)
Cancer site							
Lung	814/1018	277/426	380/443	157/149	1.27(0.63-2.61)	1.09(0.63-1.89)	1.27(0.82-1.95)
Colorectal	734/747	288/366	362/310	84/71	1.50(0.96-2.33)	1.52(0.95-2.42)	1.15(0.73-1.81)
Breast	5274/7874	2544/3848	2204/3331	526/695	1.44(0.82-2.55)	1.07(0.93-1.24)	1.15(0.73-1.81)
Other	1396/1526	641/734	621/636	134/156	1.04(0.79-1328)	1.12(0.96-1.31)	0.97(0.73-1.30)
Ethnicity							
Caucasian	5751/8345	2804/4199	2417/3463	530/683	1.29(1.27-1.63)*	1.17(1.02-1.34)*	1.19(0.98-1.45)
Asian	2253/2423	867/989	1055/1086	331/348	1.27(0.83-1.93)	1.24(0.93-1.66)	1.10(0.76-1.59)
Mixed	214/397	79/186	95/171	40/40	1.34(0.14-12.95)	0.87(0.17-4.40)	1.68(0.44-6.35)
Source of Controls							
Population	5549/7936	2590/3777	2388/3387	571/772	1.01(0.89-1.14)	1.02(0.92-1.14)	1.00(0.89-1.13)
Hospital	2556/2930	1114/1426	1128/1219	314/285	1.35(0.97-1.88)	1.21(0.98-1.50)	1.26(0.96-1.65)
Other	113/299	46/171	51/114	16/14	4.25(1.93-9.34)*	1.95(1.25-3.02)*	3.36(1.58-7.13)*

* *P*<0.05.

The characteristics of populations and cancer types of final 19 publications were listed in Table 1. This meta-analysis involved in 5 breast cancer studies, 5 lung cancer studies, 4 colorectal cancer studies and 5 studies with other cancer types. Of these, there were 10 studies conducted in Caucasian, 7 studies in Asian, 1 study in Tunisian, and 1 study included multiple ethnicities. There were 6 population-based studies, 12 hospital-based studies, and 1 study with mixed controls study. Variant genotyping methods were used, which included polymerase chain reaction restriction fragment length polymorphism assay (PCR-RFLP) in 14 studies, TaqMan assay, MassARRAY, microarray, arrayed primer extension (APEX) and unknown method in one study each. Overall, the genotyping frequencies of *CYP1A2* rs762551 polymorphism were in agreement with the Hardy-Weinberg equilibrium in both cases and controls.

### Quantitative synthesis

**Figure 2:**
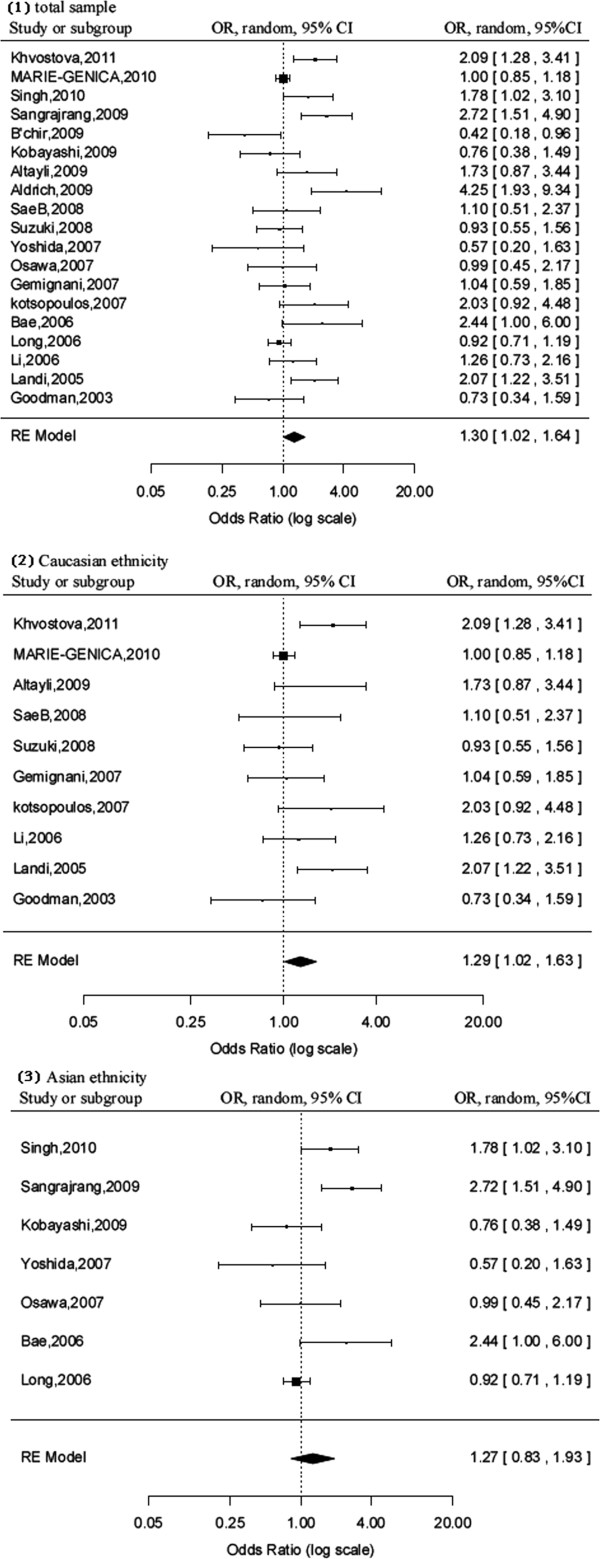
**Forest plot of cancer risk associated with *CYP1A2 *rs762551 polymorphism in different ethnicity.** Pooled odds ratio (OR) for (1) total samples, (2) Caucasians ethnicity, and (3) Asians ethnicity. The genetic models are CC versus AA. The squares and horizontal lines correspond to the study-specific odds ratio (OR) and 95% confidence interval (CI).

Regarding *CYP1A2* rs762551 polymorphism, the eligible studies involved in 8218 cases and 11165 controls. For each study, we investigated the association based on the assumption of different inheritance models of *CYP1A2* rs762551 A>C polymorphism. In all inheritance models of rs762551 polymorphism, due to the between-study heterogeneity in the individual studies (all *P* for Q test < 0.01 and I^2^>25%), the random-effect model was used to analyze the data [42]. We identified that rs762551 polymorphism had a weak correlation with the risk of cancer (CC versus AA, OR = 1.30, 95% CI = 1.02-1.64; dominant model, OR = 1.19, 95% CI = 1.04-1.36), but not in recessive model (OR = 1.19, 95% CI = 0.99-1.44) (Figure 2 and Table 2).

Several subgroup analyses were also performed according to the population ethnicity, cancer type, or source of control population (Table 2). When we analyzed the relationship of rs762551 polymorphism and cancer risk in different ethnicity subgroup. Our data showed that rs762551 A>C polymorphism increased the cancer risk (CC versus AA, OR = 1.29, 95% CI = 1.27-1.63; dominant model, OR = 1.17, 95% CI = 1.02-1.34) in Caucasians, but not in Asians (CC versus AA, OR = 1.27, 95% CI = 0.83-1.93; dominant model, OR = 1.24, 95% CI = 0.93-1.66) and in other mixed population (CC versus AA, OR = 1.34, 95% CI = 0.14-12.95; dominant model, OR = 0.87, 95% CI = 0.17-4.40). In recessive genetic model, our study didn’t show any significant correlation between rs762551 polymorphism and the cancer risk with OR (95% CI) of 1.19 (0.98-1.45), 1.10 (0.76-1.59) and 1.68 (0.44-6.35) in Caucasians, Asians and other mixed population, respectively (Figure 2 and Table 2). We didn’t observe any significant association among other subgroups (cancer type and source of control) subgroup in any genetic model using random effect model (Table 2).

### Sensitivity analysis

In order to compare the sensitivity of the meta-analysis, we conducted a leave-one-out sensitivity analysis (Additional file 1: Table S1). A single study involved in this meta-analysis was evaluated each time to reflect the influence of the individual data set to pooled ORs. The results pattern was not impacted by single study in all genetic models. The *P* for Q test and the I^2^ value also showed that none of single study affected the heterogeneity of this meta-analysis.

### Statistical uncertainty of finding

For risk assessment, statistical uncertainty is associated with the model selected [43]. In this meta-analysis, we evaluated the association of *CYP1A2* rs762551 polymorphism with cancer risk in different genetic models (CC versus AA model, dominant model and recessive model). The results showed the difference among these genetic models. We found the rs672551 polymorphism was associated with cancer risk under CC versus AA model and dominant model, but not under recessive model.

### Bias diagnostics

**Figure 3:**
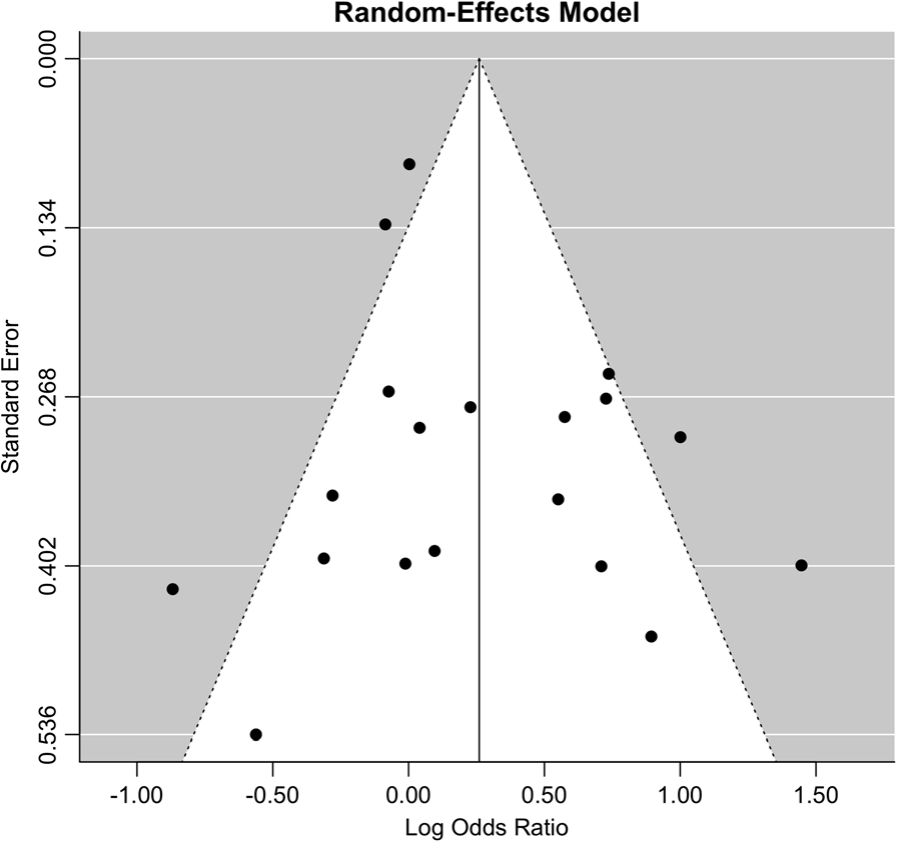
**Funnel plot analysis to detect publication bias for rs762551 polymorphism (CC versus AA).** Each point represents an individual study for the indicated association.

To evaluate the publication bias of rs762551 variant in the overall meta-analysis, the funnel plot and Egger’s test were used. In this analysis, the funnel plot showed a relatively symmetric distribution (Figure 3), but the point cloud didn’t have a distinctive form. No publication bias was detected by the Egger’s test (*t* = 1.4870, *P* = 0.1553). However, the deficient funnel form of the funnel plot could be due to the relatively high heterogeneity with respect to the different ethnicity and the source of control population. Furthermore, the number of studies was relatively small and the publication bias may still exist.

## Discussion

Various studies provided evidences that genetics play an important role in determining cancer risk and association studies have been identified to evaluate cancer susceptibility [44]. However, many association studies failed to provide convincing evidence of linkage and have resulted in contradicting findings, especially in small sample sizes [45]. Meta-analysis provided a popular method for combining world literatures across studies to resolve the statistical power and discrepancy problem in associate studies [46]. Based on 19 studies providing data on *CYP1A2* rs762551 polymorphism and cancer risk, we conduced a meta-analysis involving in 8218 cancer cases and 11165 controls to indicate if the rs762551 polymorphism was significantly associated with risk of cancer. We evaluated the publication bias. The *CYP1A2* rs762551 A>C genotypes funnel plot was approximately symmetrical and the Eggle’s test showed that there is no publication bias in the study of *CYP1A2* rs762551 (*P* = 0.1553). We found that the carriers of *CYP1A2* rs762551 C allele had a weak effect on the overall cancer risk in allele genetic model and in the dominant genetic model. These results suggested that the *CYP1A2* rs762551 polymorphism might be useful for assessing cancer risk.

CYP1A2 is a critical enzyme involved in drug metabolism and carcinogen bioactivation. The expression and activity of CYP1A2 has been demonstrated to relate to the risk of various cancers [2,6,41,47]. *CYP1A2* gene is genetically polymorphic in human. To date, 177 SNPs have been deposited in the NCBI database and the frequency of these SNPs varies by ethnicity. Many of these SNPs are in linkage disequilibrium and a few SNPs have been reported to be functional [14,48]. For example, CYP1A2*1F (rs762551) polymorphism can result in 2–3 fold increase in activity/protein and has been associated with inducibility [49]. Our current analysis showed that the CC genotype of rs762551 elevated the individual susceptibility to the cancer risk. This is consistent to the function of *CYP1A2* rs762551 polymorphism. However, we didn’t observe any correction of CC genotype with the cancer risk in subgroup analysis by cancer type. This biochemical mechanism was still unclear. In addition, due to the low OR in our study, our results should be interpreted cautiously. The *CYP1A2* polymorphism may prove to be useful for assessing cancer risk.

It has been well known that cancer occurrence and mortality varied by ethnicity and geographic location [50]. In this meta-analysis, all subjects were subgrouped into three groups (Caucasian, Asian and other populations). No association of rs762551 polymorphism with cancer risk was detected in Asian and mixed population, while increased cancer risk was demonstrated in Caucasians. This finding reflected the difference of cancer susceptibility in different ethnicity, due to different genetic background and environmental exposure. However due to the low OR in this meta-analysis, further investigation still need to be conducted in a large scale Asian population.

Any meta-analysis has it limitations. To better interpreting the finding, several limitations need to be considered in current analysis. Firstly, potential publication biases may exist in this meta-analysis because studies excluded the non-English-language publications. Secondly, the total study size was still too small to perform subgroup analysis. Thirdly, this meta-analysis was based on unadjusted data due to a lack of detailed genotype information stratified by main confounding variables, such as gender, age, smoking status in original articles.

## Conclusions

In summary, our meta-analysis demonstrated a weak association of *CYP1A2* rs762551 polymorphism with cancer risk, mainly in Caucasian population. However, as a potentially powerful tool for assessing population effects of genetic variants, meta-analysis cannot replace for adequate genetic association studies. Also, to reach a more definitive conclusion, further gene-gene interaction and gene-environment interaction studies, which based on large sample size, are still needed in different population.

## Abbreviations

CYP1A2: Cytochrome P450 1A2; OR: Odd ratio; CI: Confidence interval; HAA: Heterocyclic aromatic amines, PAHs, polycyclic aromatic hydrocarbons; NNK: 4-methylnitrosamino-1- (3-pyridyl) -1-butanone; PCR-RFLP: Polymerase chain reaction-restriction fragment length polymorphism assay; SNPs: Single nucleotide polymorphisms.

## Competing interests

The authors declare that they have no competing interests.

## Authors’ contributions

JY designed this study. HW and ZZ drafted the manuscript. HW, YL and SH contributed to the data extraction. ZZ and FF were responsible for data analysis. All authors read and approved the final manuscript.

## Pre-publication history

The pre-publication history for this paper can be accessed here:

http://www.biomedcentral.com/1471-2407/12/528/prepub

## Supplementary Material

Additional file 1 **Table S1. **ORs (95% CI) of sensitivity analysis for rs762551. Click here for file
