# Supplementary material for: CYP1A2 rs762551 polymorphism contributes to cancer susceptibility: a meta-analysis from 19 case-control studies
Source: BMC Cancer. 2012 Nov 19;12:528. doi: 10.1186/1471-2407-12-528 (PMC3526566; doi:10.1186/1471-2407-12-528)
Supplement: Additional file 1 — Table S1. ORs (95% CI) of sensitivity analysis for rs762551. [file 1471-2407-12-528-S1.doc]

Table S1. ORs (95% CI) of sensitivity analysis for rs762551

| Excluding literature  one by one | C allele versus A allele | | CC versus AA | | Dominant model  (CC+AC versus AA) | | Recessive model  (CC versus AC+AA) | |
| --- | --- | --- | --- | --- | --- | --- | --- | --- |
|  | OR (95% CI) | *P*h, I^2^ | OR (95% CI) | *P*h, I^2^ | OR (95% CI) | *P*h, I^2^ | OR (95% CI) | *P*h, I^2^ |
| All for rs762551 | 1.14(1.04-1.26) | <0.01, 67.5 | 1.30(1.02-1.64) | <0.01, 67.5 | 1.19(1.04-1.36) | <0.01, 67.5 | 1.19(0.99-1.44) | <0.01, 67.5 |
| Khvostova 2011 | 1.13(1.02-1.24) | <0.01, 72.5 | 1.26(0.99-1.60) | <0.01, 72.5 | 1.17(1.02-1.35) | <0.01, 72.5 | 1.16(0.96-1.40) | <0.01, 72.5 |
| MARIE-GENICA 2010 | 1.16(1.05-1.28) | <0.01, 67.5 | 1.33(1.03-1.71) | <0.01, 67.5 | 1.19(1.03-1.40) | <0.01, 67.5 | 1.21(0.99-1.49) | <0.01, 67.5 |
| Singh 2009 | 1.13(1.02-1.25) | <0.01, 74.3 | 1.27(0.99-1.63) | <0.01, 74.3 | 1.22(1.08-1.35) | <0.01, 74.3 | 1.19(0.97-1.45) | <0.01, 74.3 |
| Sangrajrang 2009 | 1.13(1.02-1.25) | <0.01, 69.7 | 1.24(0.99-1.56) | <0.01, 69.7 | 1.19(1.03-1.38) | <0.01, 69.7 | 1.14(0.96-1.34) | <0.01, 69.7 |
| B´chir 2009 | 1.16(1.06-1.27) | <0.01, 68.7 | 1.36(1.09-1.70) | <0.01, 68.7 | 1.18(1.03-1.37) | <0.01, 68.7 | 1.21(1.00-1.48) | <0.01, 68.7 |
| Kobayashi 2009 | 1.15(1.04-1.27) | <0.01, 73.3 | 1.33(1.05-1.70) | <0.01, 73.3 | 1.16(1.02-1.37) | <0.01, 73.3 | 1.23(1.02-1.48) | <0.01, 73.3 |
| Altayli 2009 | 1.13(1.03-1.25) | <0.01, 74.7 | 1.28(1.00-1.64) | <0.01, 74.7 | 1.19(1.03-1.36) | <0.01, 74.7 | 1.18(0.97-1.44) | <0.01, 74.7 |
| Aldrich 2009 | 1.11(1.021.22) | <0.01, 66.4 | 1.23(0.99-1.53) | <0.01, 66.4 | 1.21(1.04-1.32) | <0.01, 66.4 | 1.14(0.96-1.36) | <0.01, 66.4 |
| Saebo 2008 | 1.14(1.03-1.26) | <0.01, 75.1 | 1.31(1.02-1.68) | <0.01, 75.1 | 1.20(1.05-1.37) | <0.01, 75.1 | 1.21(0.99-1.47) | <0.01, 75.1 |
| Suziki 2008 | 1.15(1.04-1.28) | <0.01, 74.1 | 1.33(1.03-1.70) | <0.01, 74.1 | 1.21(1.06-1.39) | <0.01, 74.1 | 1.21(1.00-1.48) | <0.01, 74.1 |
| Yoshida 2007 | 1.15(1.05-1.27) | <0.01, 73.2 | 1.33(1.05-1.69) | <0.01, 73.2 | 1.18(1.02-1.38) | <0.01, 73.2 | 1.22(1.01-1.47) | <0.01, 73.2 |
| Osawa 2007 | 1.15(1.04-1.27) | <0.01, 74.8 | 1.31(1.03-1.68) | <0.01, 74.8 | 1.20(1.04-1.38) | <0.01, 74.8 | 1.20(0.98-1.46) | <0.01, 74.8 |
| Gemignani 2007 | 1.14(1.03-1.26) | <0.01, 74.8 | 1.31(1.02-1.69) | <0.01, 74.8 | 1.15(1.03-1.26) | <0.01, 74.8 | 1.22(1.00-1.48) | <0.01, 74.8 |
| Kotsopoulos 2007 | 1.14(1.03-1.26) | <0.01, 74.1 | 1.27(1.00-1.62) | <0.01, 74.1 | 1.21(1.04-1.38) | <0.01, 74.1 | 1.17(0.97-1.41) | <0.01, 74.1 |
| Bae 2006 | 1.12(1.02-1.23) | <0.01, 73.5 | 1.27(1.00-1.61) | <0.01, 73.5 | 1.18(1.02-1.29) | <0.01, 73.5 | 1.20(0.98-1.46) | <0.01, 73.5 |
| Long 2006 | 1.16(1.04-1.28) | <0.01, 70.1 | 1.33(1.04-1.71) | <0.01, 70.1 | 1.17(1.02-1.40) | <0.01, 70.1 | 1.23(1.01-1.50) | <0.01, 70.1 |
| Li 2006 | 1.14(1.03-1.26) | <0.01, 75.1 | 1.30(1.01-1.67) | <0.01, 75.1 | 1.20(1.05-1.37) | <0.01, 75.1 | 1.20(0.98-1.47) | <0.01, 75.1 |
| Landi 2005 | 1.13(1.02-1.24) | <0.01, 72.9 | 1.26(0.99-1.61) | <0.01, 72.9 | 1.21(1.04-1.35) | <0.01, 72.9 | 1.17(0.96-1.41) | <0.01, 72.9 |
| Goodman 2003 | 1.15(1.04-1.27) | <0.01, 73.5 | 1.33(1.05-1.70) | <0.01, 73.5 | 1.20(1.05-1.39) | <0.01, 73.5 | 1.22(1.01-1.48) | <0.01, 73.5 |
